# Novel Neurocognitive Testing Tool for Early Neurotoxicity Detection Following Anti-CD19 and Anti-BCMA Chimeric Antigen Receptor (CAR) T-cell Therapy: A Pilot Study

**DOI:** 10.1016/j.clml.2024.12.011

**Published:** 2024-12-24

**Authors:** Arvind Suresh, Heather A. Wishart, Maeen N. Arslan, Raphael A. Lizcano, Parth S. Shah, Swaroopa PonnamReddy, Christi Ann Hayes, Bryce S. Jacobson, Grant Moncrief, Pablo Martinez-Camblor, Amy M. Chan, Kenneth R. Meehan, John M. Hill

**Affiliations:** 1 Department of Medicine, University of California, San Francisco, CA; 2 Department of Psychiatry, Dartmouth-Hitchcock Medical Center, Lebanon, NH; 3 Dartmouth College, Hanover, NH; 4 Transplant and Cellular Therapy Program, Dartmouth Cancer Center, Dartmouth-Hitchcock Medical Center, Lebanon, NH; 5 Department of Biomedical Data Science, Geisel School of Medicine at Dartmouth, Lebanon, NH

**Keywords:** ICANS, Biomarkers, Early detection

## Abstract

We assessed the utility of a rapid neurocognitive testing tool in the early detection of ICANS and its ability to predict which patients will develop ICANS following CAR T-cell therapy. We found that the Oral Symbol Digit, Stroop, and Paced Visual Serial Addition tests were sensitive and specific in predicting ICANS development and better capture the full spectrum of neurotoxicity.

**Background:**

Immune effector cell-associated neurotoxicity syndrome (ICANS) can be a severe, life-threatening toxicity following CAR T-cell therapy. While currently evaluated by the immune effector cell-associated encephalopathy (ICE) score, not all patients have changes in their ICE score and not all signs and symptoms of neurotoxicity are captured.

**Methods:**

We conducted a prospective, single center cohort pilot study to evaluate a novel, rapid neurocognitive assessment tool (CART-NS) in detecting early, subtle neurotoxicity prior to the onset of ICANS and any deterioration in the ICE score. CART-NS includes 8 abbreviated forms of neurocognitive tests and 2 symptom questionnaires. Following baseline measurements, CART-NS was administered at 8-hours intervals during the first 30 days after CAR T-cell infusion.

**Results:**

Performance on all measures was significantly lower when patients developed Grade 1 or 2 ICANS (*P* < .05). Performance on Oral Symbol Digit, Stroop, and the Paced Visual Serial Addition Test was lower between Day 0 and +3 in patients who developed ICANS and persisted even after clinical resolution. Early changes in the Stroop test (AUC = 0.857, 95% CI 0.628–1.000) were most predictive of ICANS onset when measured during the first 36 hours following CAR T-cell infusion. Significant elevations in CRP, G-CSF, GM-CSF, IFNγ, IL-10, IL-15, IL-27, and MIG/CXCL-9 were associated with ICANS development.

**Conclusion:**

Brief neurocognitive testing can be feasibly applied for the early detection of ICANS after CAR T-cell therapy, predict which patients may go on to develop ICANS in the first 30 days, and overcome limitations of the ICE assessment tool.

## Introduction

Chimeric antigen receptor (CAR) T-cell therapy has transformed treatment of relapsed/refractory hematologic malignancies. However, it carries the risk of significant adverse events, most notably cytokine release syndrome (CRS) and neurotoxicity. Immune effector cell-associated neurotoxicity syndrome (ICANS) can be a severe, life-threatening toxicity following CAR T-cell therapy and is characterized by a wide range of clinical features and neuropsychiatric symptoms.^1^ In 2018, the American Society for Transplantation and Cellular Therapy (ASTCT) published a consensus grading scale in an effort to harmonize existing definitions and grading schemes of ICANS. This scale included neurotoxicity domains of encephalopathy as reported by the immune effector cell-associated encephalopathy (ICE) score, depressed consciousness, seizure, motor findings, and cerebral edema.^2^

The existing neurologic scales, while not entirely concordant, have effectively identified patients with clinically relevant neurotoxicity warranting aggressive treatment. However, certain neurologic and cognitive-behavioral signs, such as headache, executive disorders, confusion, bradyphrenia, personality changes, tremors, abnormal movements, hallucinations, and hemineglect, are not included.^3,4^ Accordingly, recent reports in the literature of patients undergoing CAR T-cell therapy have confirmed that early, subtle signs of neurotoxicity, up to 30 hours prior to a decline in the ICE score, are not captured by existing grading criteria.^3–9^ At our medical center, clinicians have also reported alterations from baseline neurologic status (including confusion, subjective complaints, subtle personality changes, nystagmus, and anisocoria on full neurologic exam) that did not meet ASTCT ICANS criteria. Beyond the use of grading scales, correlative biomarkers (eg, ferritin, LDH, IL-2, IL-5) have also been identified that are predictive of severe neurotoxicity and adverse survival.^10–12^

Many patients who are treated for ICANS successfully recover, but high-grade neurotoxicity carries the potential for devastating outcomes, including death, and highlights the need for early recognition.^5^ Further, early detection and intervention of ICANS may also minimize the need for extended high-dose steroids, related morbidity, and potential impact on CAR T-cell efficacy.^13^ There remains a need to identify patients at greatest risk of severe ICANS and the patients likely to experience long-lasting neuropsychiatric effects.^1^ As detection of neurotoxicity by grading criteria is used to guide therapy, more refined assessments to capture subtle changes in cognitive-behavioral and neurologic function that currently fall outside of the ICE/ICANS grading system may lead to earlier intervention. In the current study, we present findings from the administration of abbreviated neurocognitive testing in patients undergoing CAR T-cell therapy. First, we aimed to assess the efficacy of targeted neurocognitive testing in identifying early, subtle signs of neurotoxicity when administered in a standardized manner by clinicians or nurses at the bedside. We then assessed whether changes in the levels of serum biomarkers are associated with the development of ICANS.

## Methods

We conducted a prospective, single center cohort study to evaluate a novel rapid neurocognitive assessment tool (CART-NS) in patients undergoing CAR T-cell therapy at Dartmouth Cancer Center in Lebanon, NH between August 2021 and March 2023. Suitability for the study was determined based on standard precellular therapy criteria. All patients underwent formal neurologic testing and evaluation by a neuro-oncologist to screen for baseline neurologic abnormalities. Patients with CNS disease, history of CVA, and seizure disorders were excluded from the study. This study obtained written informed consent for all patients and was approved by the Dartmouth Health Institutional Review Board as STUDY02001042.

### Creation of Neurocognitive Assessment Tool

To identify the most appropriate neurocognitive tests for detecting neurotoxicity, we identified a set of 19 signs and symptoms that together represent commonly reported neurologic and cognitive changes following CAR T-cell therapy ( Table 1 ). Subsequently, in collaboration with neuro-oncology and neuropsychology experts at our center, we mapped these signs and symptoms to relevant neurocognitive domains previously studied in patients with neurotoxicity after CAR T-cell therapy and selected 8 neurocognitive tests that would either directly or indirectly detect these signs and symptoms (Table 1).^14,15^ The 8 neurocognitive measures we identified include the Oral Symbol Digit (ODT), Trail Making (Trails), Stroop, Paced Visual Serial Addition (PVSAT), Finger Tapping, Connecting Dots, Intersecting Figures, and Verbal Fluency. These measures were selected based on ease of administration with minimal training and relatively low susceptibility to practice effects.^16^ In collaboration with neuropsychologists, we developed abbreviated forms of each neurocognitive test as well as a patient symptom questionnaire (PSQ) and caregiver symptom questionnaire (CSQ) to create a neurocognitive assessment tool named the CAR T-cell Neurotoxicity Screener (CART-NS). Total average completion time of the full assessment tool was under 10 minute. About 3 equivalent forms of our assessment tool were created to minimize practice effects seen with the ICE score assessment while maintaining adequate reproducibility. In comparison to the Montreal Cognitive Assessment (MoCA) rapid cognitive screening tool, CART-NS is designed to assess domains specific to neurotoxicity after CAR T-cell therapy. While both tools assess memory, executive function, attention, language, and visuospatial abilities, CART-NS also tests processing speed and fine motor skills and includes 2 symptom questionnaires. Unlike the MoCA, there is no maximum score on CART-NS to facilitate serial longitudinal monitoring of bidirectional changes in performance. Pilot testing of all 3 forms was conducted with members of the research team, transplant and cellular therapy (TCT) nurses, and select patients to evaluate feasibility of test administration, clarity of instructions, and reproducibility of results. Feedback was incorporated into final versions of the test forms. Detailed descriptions of all neurocognitive tests and copies of the PSQ and CSQ are included in the Supplemental Material.

### Administration of Neurocognitive Testing

All patients underwent baseline testing with CART-NS prior to CAR T-cell infusion. Patients completed each of the 3 test forms once as a baseline during the course of lymphodepletion therapy between Day −5 and Day 0. During the first 14 days after CAR T-cell infusion, inpatient nurses administered CART-NS every 8 hours in parallel with administration of the ICE score assessment. The 3 test forms were administered to patients in a predetermined random order that was the same for all patients, with no forms repeated consecutively. If any changes were noted in the patient’s ICE score, CART-NS and ICE score testing frequency were increased to every 4 hours, in alignment with standard operating procedures at our medical center. Following hospital discharge, CART-NS and the ICE score assessment were administered once daily to patients in the outpatient setting between Day + 15 and Day + 30.

### Assessment of Predictive Biomarkers of Neurotoxicity

Patients underwent blood sample collection and plasma processing on Day 0 prior to CAR T-cell infusion, Day + 3, and Day + 7. These time points were chosen as they correspond with the mean time to development of neurotoxicity after CAR T-cell therapy.^1,6,7^ A MILLIPLEX human cytokine/growth factor 48-plex panel was performed on all patient specimens, testing for the following analytes: sCD40L, EGF, Eotaxin, FGF-2, Flt-3 ligand, Fractalkine, G-CSF, GM-CSF, GRO-alpha, IFN-alpha 2, IFN-gamma, IL-1 alpha, IL-1 beta, IL-1r alpha, IL-2, IL-3, IL-4, IL-5, IL-6, IL-7, IL-8, IL-9, IL-10, IL-12 (p40), IL-12 (p70), IL-13, IL-15, IL-17A, IL-25, IL-17F, IL-18, IL-22, IL-27, IP-10, MCP-1, MCP-3, M-CSF, MDC, MIG, MIP-1 alpha, MIP-1 beta, PDGF-AA, PDGF-AB, RANTES, TGF-alpha, TNF-alpha, TNF-beta, VEGF-A [Millipore Sigma, USA]. This 48-plex assay was chosen based on its inclusion of cytokines previously found to be associated with post-CAR T-cell neurotoxicity.^10–12,17–19^ The concentration of each of these cytokines was recorded in picograms/milliliter. Measurements of platelet count, serum creatinine, LDH, CRP, fibrinogen, and ferritin on Day 0, Day + 3, and Day + 7 were additionally extracted through chart review.

### Statistical Analysis

Changes in performance on each neurocognitive testing measure were calculated as a percentage change from mean baseline score prior to CAR T-cell infusion, with each patient serving as their own control. Pooled statistical analyses were subsequently conducted on all test administrations of CART-NS across 12 patients, grouped by the presence or absence of clinically relevant ICANS, as defined by ASTCT criteria.^2^ Independent 2-tailed t-tests with Welch’s correction were performed to compare mean scores for each CART-NS neurocognitive measure between patients who did and did not develop ICANS, as well as patients who did and did not have ICE score changes. Cohen’s d was calculated as a measure of effect size between patient groups.^20^ Significant differences in test performance between patients with and without ICANS on each day following CAR T-cell infusion were determined using independent 2-tailed t-tests with Welch’s correction, while a repeated measures ANOVA with Tukey’s multiple comparisons test was utilized to determine significant differences in test performance compared to baseline and previous timepoints. A 1-way ANOVA with Tukey’s multiple comparisons test was used to compare biomarker levels for various time points and patient groups. We created receiver operating characteristic (ROC) curves for select neurocognitive measures that appeared to significantly differ between patients with and without ICANS using the lowest performance scores during the first 36 hours after CAR T-cell infusion for each patient. Respective areas under the curve (AUC) were calculated for each ROC curve with 95% confidence intervals (CI) to summarize predictive potential. A p-value of less than .05 was used for statistical significance and a d value of greater than 0.8 was used to determine large effect sizes.^21^ SPSS version 27.0^22^ was used for all statistical analyses.

## Results

### Patient Characteristics and Incidence of ICANS and CRS

**Figure 1:**
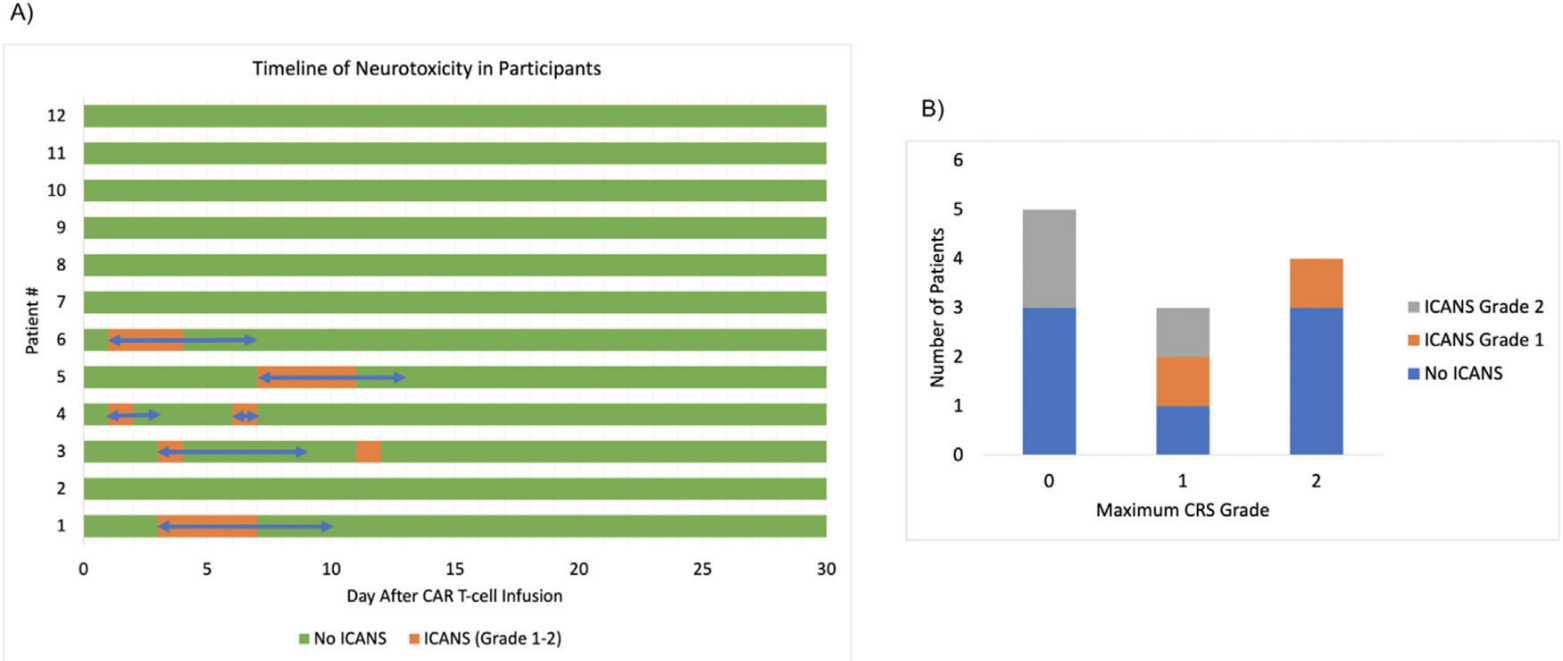
Timeline of Neurotoxicity Development and Association with CRS. (A) is a swimmer plot with orange demonstrating days with documented ICANS for each patient. The blue arrow indicates the duration of treatment with dexamethasone. The median time to onset of neurotoxicity was Day + 3 and the median duration of neurotoxicity was 3 days. No patients in the study developed severe (Grade 3 or 4) neurotoxicity per ASTCT criteria. (B) displays the distribution of CRS in study patients and which patients also developed ICANS.

About 12 patients underwent CART-NS testing during the first 30 days following CAR T-cell infusion and biomarker sample collection on Day 0, Day + 3, and Day + 7. No patients had CNS involvement in their malignancy, history of stroke, or any other neurologic comorbidities. 75% of participants were male and 25% were female. The median age at the time of CAR T-cell infusion was 65. About 4 patients each received axicabtagene ciloleucel, tisagenlecleucel, and idecabtagene vicleucel. Additional demographics and characteristics of study participants are listed in Table 2. All but 2 patients received bridging therapy prior to CAR T-cell infusion and only 1 patient was in a complete remission at the time of CAR T-cell therapy. Figure 1A demonstrates the timeline of neurotoxicity for each patient in a swimmer plot, and Figure 1B demonstrates the overlap between CRS and ICANS development in patients. Of the 12 study participants, 5 patients developed ICANS, with 2 patients developing Grade 1 and 3 patients developing Grade 2. The median time to onset of ICANS was Day + 3 with clinical resolution in all patients by Day + 11. The median peak C-reactive protein (CRP) level during the first 30 days in patients who developed ICANS was 89.3 (range 47.5–126.5), compared with a CRP of 42.1 (range 12.9–134.2) in patients without ICANS. Notably, 1 patient met ASTCT criteria for ICANS after developing a depressed level of consciousness without any changes in ICE score during the first 30 days after CAR T-cell therapy. No patients developed severe (Grade 3 or 4) ICANS. All patients who developed any grade ICANS underwent EEG monitoring. No patients had evidence of seizure activity, but EEG changes consistent with encephalopathy, including generalized delta slowing or bursts of generalized rhythmic delta activity, were identified in 3 out of 5 patients who developed ICANS. All Grade 1 CRS was treated with tocilizumab, and Grade 1 ICANS was treated with dexamethasone, in accordance with previously established center protocols. Patients were treated with low-dose, short-term dexamethasone between 3 and 7 days, with a taper when necessary, depending on the severity of ICANS. No patients received prophylaxis with dexamethasone or anakinra prior to the onset of ICANS.

### Performance in Neurocognitive Measures Differs During the Presence of ICANS and ICE Score Changes

CART-NS was administered to patients a total of 862 times in the study, divided between 37 baseline measurements and 825 measurements following CAR T-cell therapy. All patients in the study underwent baseline testing with all 3 versions of CART-NS during the week prior to CAR T-cell infusion. Following product infusion on Day 0, patients underwent parallel testing with both the ICE assessment and CART-NS every 4 to 8 hours between Day 0 and Day +14, then daily between Day +15 and Day +30 outpatient visits. Table 3 compares overall patient performance on each of the tests comprising CART-NS between timepoints when patients had ICANS (*n* = 107) and when they did not (*n* = 718), as well as timepoints when patients had ICE score changes (*n* = 28) and when they did not (*n* = 797). Scores for each test except the symptom questionnaires were normalized to each individual’s baseline performance and are displayed as a percentage increase or decrease in performance. Positive percentages indicate an improvement in performance, and negative percentages indicate a decline in performance. Mean scores for most CART-NS measures significantly differed during timepoints when patients developed ICANS and/or ICE score changes, and all patients who developed ICANS had decreased performance in 1 or more CART-NS measures. During timepoints when patients developed Grade 1 or 2 ICANS, performance on OSD, Trails, Stroop, PVSAT, Connecting Dots, Intersecting Figures, and Verbal Fluency was significantly worse, and more symptoms were reported on the PSQ and CSQ. Compared to timepoints without ICANS, scores changed by −12% on OSD (vs. +13%, *P* < .001), −29% on Trails (vs. +17%, *P* < .001), −21% on Stroop (vs. + 15%, *P* < .001), and between −25% (vs. −12%, *P* = .002) and −39% (vs. −18%, *P* = .005) on Connecting Dots, on average. Although scores did not noticeably decrease on PVSAT (+6% vs. +24%, *P* = .014) and Verbal Fluency (+0% vs. + 17%, *P* < .001), a significant group difference in performance was still seen, most likely due to differential practice effects. These effects led to a noticeable increase in test performance in patients who did not develop ICANS or ICE score changes. During timepoints when patients developed ICE score changes (ICE < 10), performance on OSD, Trails, Stroop, PVSAT, Finger Tapping, and Verbal Fluency was significantly worse, and more symptoms were reported on the CSQ. Compared to timepoints with an ICE score of 10, scores changed by −35% on OSD (vs. +12%, *P* < .001), −117% on Trails (vs. +15%, *P* < .001), −36% on Stroop (vs. +12%, *P* < .001), and −5% on PVSAT (vs. +23%, *P* < .001). Performance on Finger Tapping and Verbal Fluency differed due to practice effects in patients without ICE score changes. Changes across multiple tests and domains occurred simultaneously when patients developed Grade 1 ICANS. Trails (d = 0.92), Stroop (d = 0.80), and CSQ (d = 1.37) had the largest degree of change in patients during the incidence of ICANS, while Finger Tapping (d = 0.02 and 0.29), Intersecting Figures (d = 0.38), and Connecting Dots (d = 0.34 and 0.31) had the smallest.

### Neurocognitive Measures Detect Early and Delayed Neurotoxicity in Patient with ICANS

**Figure 2:**
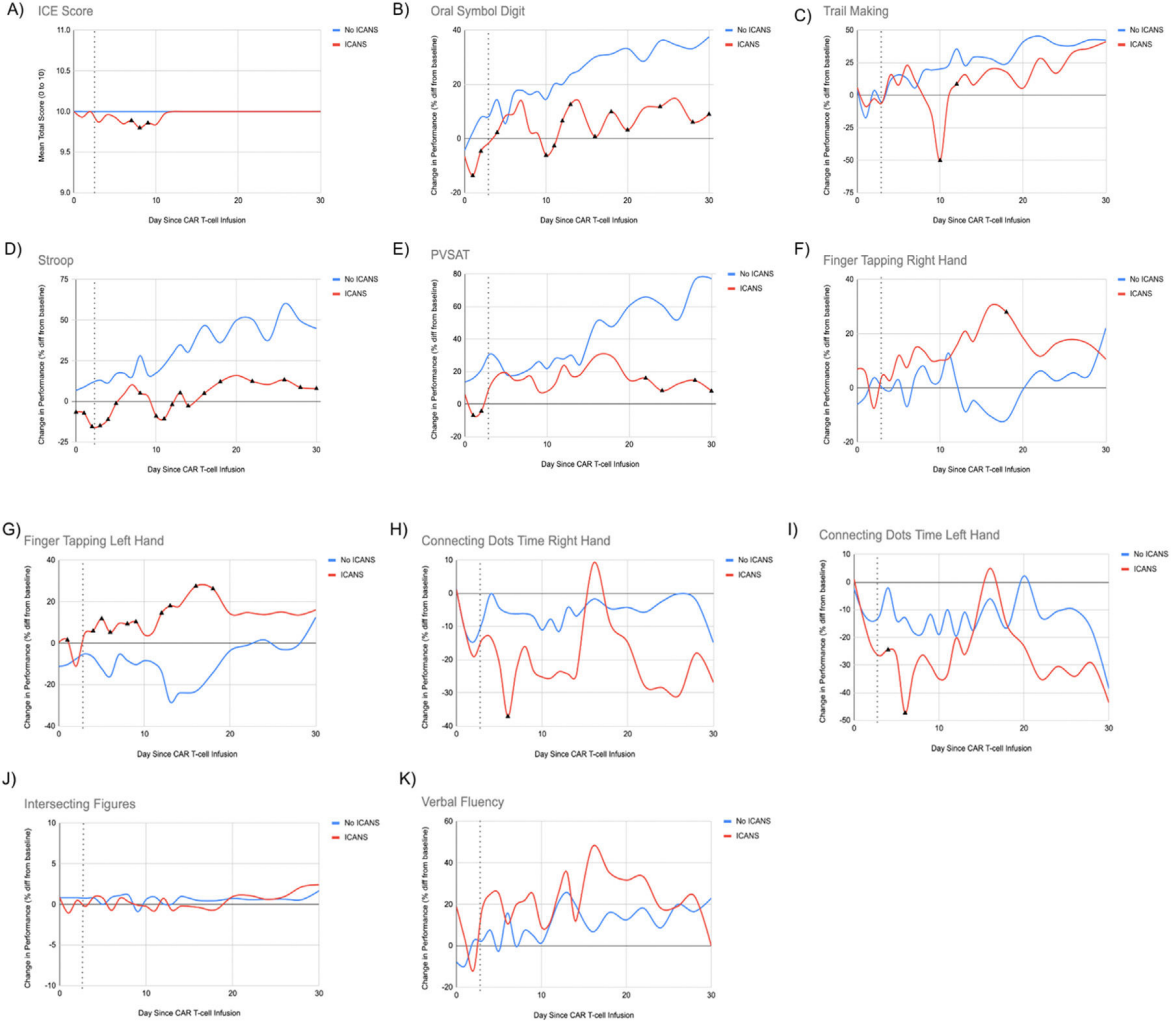
Changes in neurocognitive measures after CAR T-cell therapy in patients with and without ICANS. (A) demonstrates changes in the ICE score on a scale of 0–0. The dotted line indicates median onset of ICANS in the study sample (Day +3). (B-K), scores for each subsequent neurocognitive measure are reported as mean percent change from baseline scores for each day beginning with the day of CAR T-cell infusion (Day 0). Scores above 0 indicate better performance and scores below 0 indicate worse performance compared with baseline scores. Black triangles indicate days with significant differences in performance between patients who had ICANS and patients who did not (*P* < .05). * All patients except 1 in each subgroup (ICANS and non-ICANS) were right-hand dominant.

To further identify the ability of each neurocognitive measure in CART-NS to detect neurologic changes in patients with ICANS, we compared the mean test performance between patients who did and did not develop ICANS during each day following CAR T-cell infusion. Figure 2 demonstrates changes in mean performance in each test for these 2 patient groups plotted over time, with negative numbers indicating a decline in performance from baseline and positive numbers indicating an improvement in performance. Black triangles mark significant differences between patients with and without ICANS for each day. In patients who developed ICANS, performance on OSD, Stroop, and PVSAT was significantly lower between Day 0 and Day +3, the median time to onset of ICANS in the study sample. OSD performance was 16% lower on Day +1 (*P* = .033), 12% lower on Day +2 (*P* = .014), and 10% lower on Day + 3 (*P* = .096) in patients who developed ICANS and improved by Day +4 to baseline performance. Notably, however, performance remained different between the 2 groups until Day + 30, as patients with ICANS did not have improvement in performance due to repeated practice. Similar changes were seen with Stroop performance, which was 16% lower on Day +1 (*P* = .013), 27% lower on Day +2 (*P* < .001), and 28% lower on Day +3 (*P* < .001) but improved by Day +6. Despite this, significant differences in Stroop performance remained until Day + 30 due to an absence of practice effects in ICANS patients. PVSAT performance was 23% lower on Day +1 ( *P* = .005), 25% lower on Day +2 ( *P* = .001), and 20% lower on Day +3 ( *P* = .059), improving back to baseline by Day +3. The absence of practice effects in ICANS patients seen with OSD, Stroop, and PVSAT was not seen with the other tests. For instance, with Trails, both patients with and without ICANS improved their baseline score by 42% and 41% by Day +30, respectively. Trails also demonstrated a delayed trough in performance on Day +10, which was 70% lower in patients with ICANS (*P* = .011), but quickly recovered in subsequent days. Together, these findings demonstrate the wide spectrum of neurotoxicity that can persist beyond recovery of the ICE score and clinical resolution of ICANS. Performance on Finger Tapping paradoxically increased in patients with ICANS for both the right and left hand, which was not seen on the other measures. Performance on Connecting Dots reached a trough on Day +6 for both the right and left hand, with other time points not significantly different between the 2 groups due to a greater variability in response time. Intersecting Figures and Verbal Fluency both did not demonstrate any significant differences between patients with and without ICANS on any of the days tested following CAR T-cell therapy. In the subset of patients who developed ICANS, we also evaluated how closely these changes in neurocognitive measures occurred relative to the onset of ICANS by standard ASTCT criteria (see Supplemental Figure S1). Compared to the ICE score, for which performance changed only on the day of ICANS onset (10 vs. 9.67, *P* = .008), trends in decreasing performance in the days leading up to ICANS onset are seen with OSD, Stroop, and Connecting Dots. These changes in performance prior to ICANS onset were not statistically significant given the smaller subset of patients analyzed.

### Patient and Caregiver Reported Symptom Burden Coincides with Presence of ICANS

**Figure 3:**
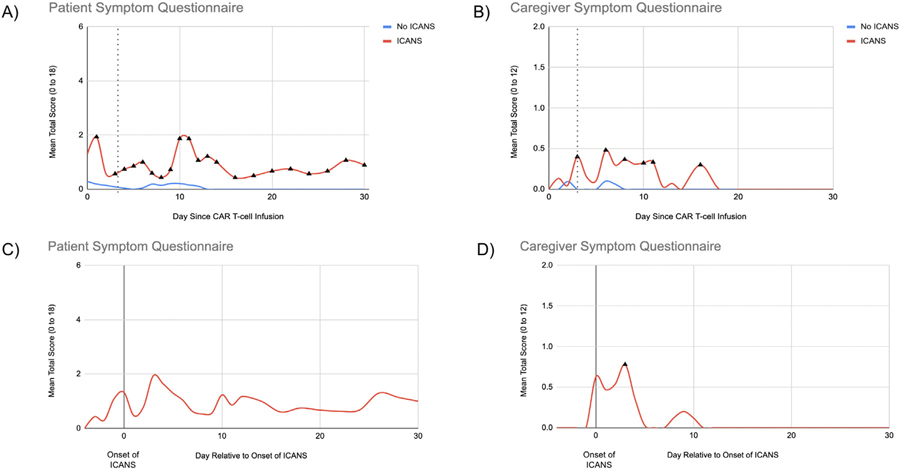
Changes in patient and caregiver reported symptom burden. The Patient Symptom Questionnaire (PSQ) is scored from 0 to 18 and the caregiver symptom questionnaire is scored from 0 to 12, with higher scores indicating greater symptom burden. (A and B) show mean scores on the PSQ and CSQ for each day after CAR T-cell infusion in patients who developed ICANS and patients who did not. The dotted line indicates median onset of ICANS in the study sample (Day +3). Black triangles indicate days where there were significant differences in mean scores between patients with and without ICANS (*P* < .05). (C and D) show mean total scores on the PSQ and CSQ relative to the onset of neurotoxicity in patients who developed ICANS. Days to the left of 0 are prior to the onset of ICANS and days to the right of 0 are after the onset of ICANS. Black triangles indicate days with a significant change in reported symptom burden compared to baseline ( *P* < .10).

Changes in symptom burden on the PSQ and CSQ were evaluated in patients with and without ICANS during each day following CAR T-cell infusion using a similar methodology as with the neurocognitive measures. For each day after infusion, the mean score on each symptom questionnaire was compared between patients with and without ICANS, as displayed in Figure 3. Patients who developed ICANS reported a significantly greater symptom burden on the PSQ during the 30 days following CAR T-cell therapy. When compared to patients who did not develop ICANS, patients with ICANS had the greatest mean PSQ scores on Day +1 (1.93vs. 0.19, *P* = .024), Day +10 (1.87vs. 0.21, *P* = .012), and Day +11 (1.87vs. 0.16, *P* < .001). Relative to the onset of ICANS, most patients reported symptoms on the day of ICANS onset and 3 days afterwards. The most reported symptoms were difficulty concentrating, followed by new anxiety, new headache, and new depression or loss of interest in activities. About 1 patient reported strange smells and flashing lights beginning on Day +10 following CAR T-cell therapy that improved by Day +12. On the CSQ, nurses who completed the questionnaire also observed a significantly greater symptom burden in ICANS patients compared to patients without ICANS, with the greatest mean scores on Day +3 (0.4 vs. 0, *P* = .031), Day +6 (0.48 vs. 0.1, *P* = .038), and Day +8 (0.37 vs. 0, *P* = .046). Relative to the onset of ICANS, most caregivers reported symptoms on the day of ICANS onset and 3 days thereafter. The most common observations reported on the CSQ were new confusion in activities of daily living, followed by new speech difficulty, restlessness and mood changes. In the free text response section, nurses reported additional observations not directly queried, including word-finding difficulty, slower response time, lethargy, unsteady gait, hand tremor, and fumbling with objects.

### Early Changes in Neurocognitive Measures Accurately Predict Incidence of ICANS

**Figure 4:**
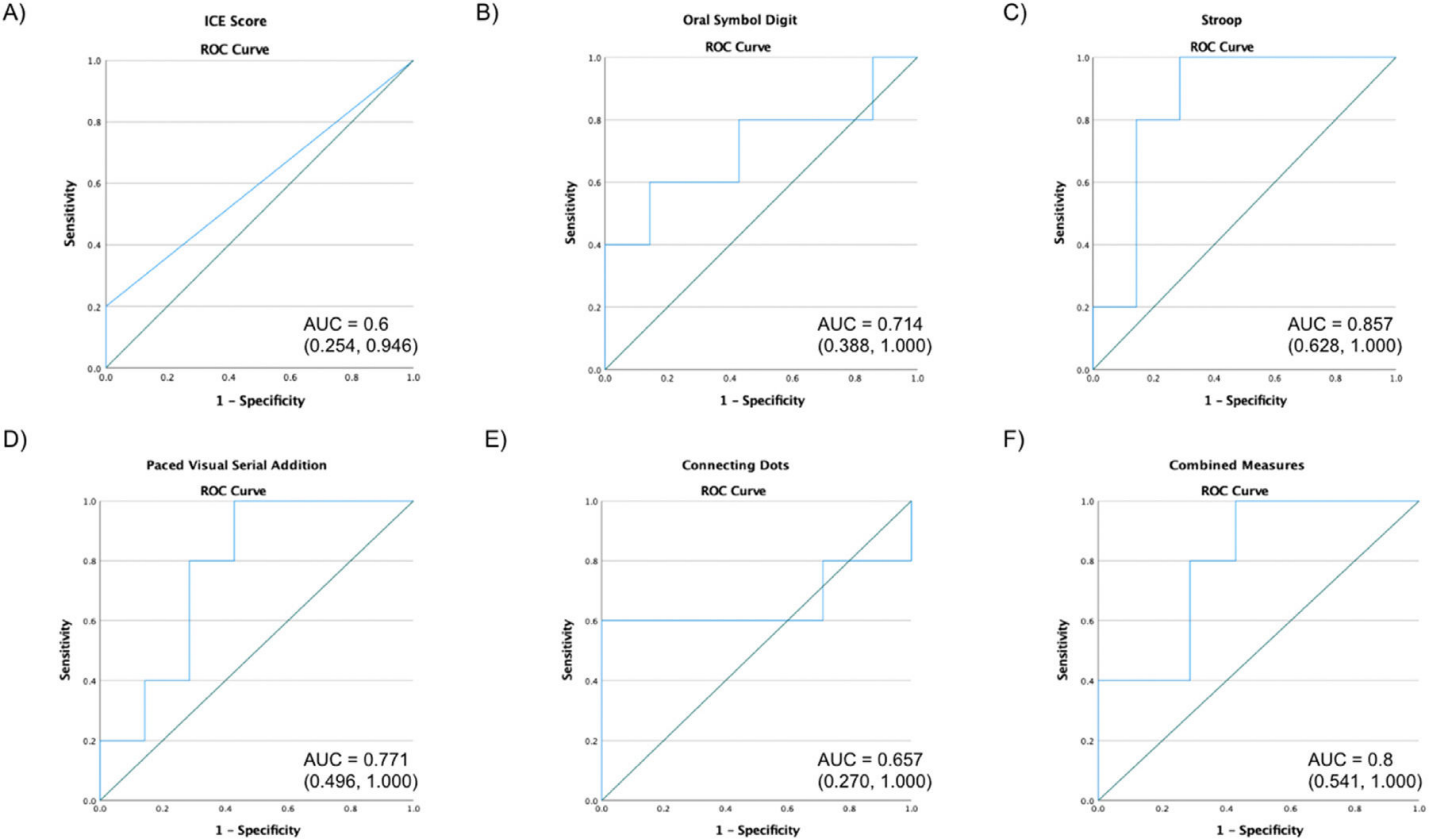
Receiver Operating Characteristic Curves for Neurocognitive Measures. (A-F) demonstrate receiver operating characteristic (ROC) curves for the ICE score and select neurocognitive measures in CART-NS to assess their predictive value for ICANS.

To assess the predictive value of the tests with the greatest differences in performance between patients with and without ICANS, receiver operating characteristic (ROC) curves were created for the ICE score, OSD, Stroop, PVSAT, and Connecting Dots. The lowest performance score for each test during the first 36 hours following CAR T-cell infusion was used to predict the occurrence of ICANS in all study participants. The first 36 hours were chosen, since most patients developed ICE score changes after this time point. Thirty-six hours corresponds to the end of Day + 1 due to patients having received their product infusion at noon on Day 0. Areas under the curve (AUC) were 0.714 for OSD (95% CI 0.388–1.000), 0.857 for Stroop (95% CI 0.628–1.000), 0.771 for PVSAT (95% CI 0.496–1.000), and 0.657 for Connecting Dots (95% CI 0.270–1.000), as shown in Figure 4 . A combined measure with the sum of the lowest scores of the OSD, Stroop, and PVSAT had an AUC of 0.8 (95% CI 0.541–1.000). All 4 tests and the combined measure had a higher AUC when compared with the ICE score (AUC = 0.6, 95% CI 0.254–0.946).

For Figure 4 A, the lowest ICE score in the first 36 hours after CAR T-cell infusion was used to predict whether or not patients would develop ICANS.

For Figure 4 B-E, the lowest performance on each single neurocognitive test, measured as a percentage decline from baseline scores for each individual in the first 36 hours after CAR T-cell infusion, was used to predict ICANS.

For Figure 4 F, a combined measure of the lowest sum of scores from the Oral Symbol Digit, Stroop, and Paced Visual Serial Addition tests in the first 36 hours after CAR T-cell infusion was used to predict ICANS.

### Serial Measurements of Serum Biomarkers Differ Between Patients With and Without ICANS

**Figure 5:**
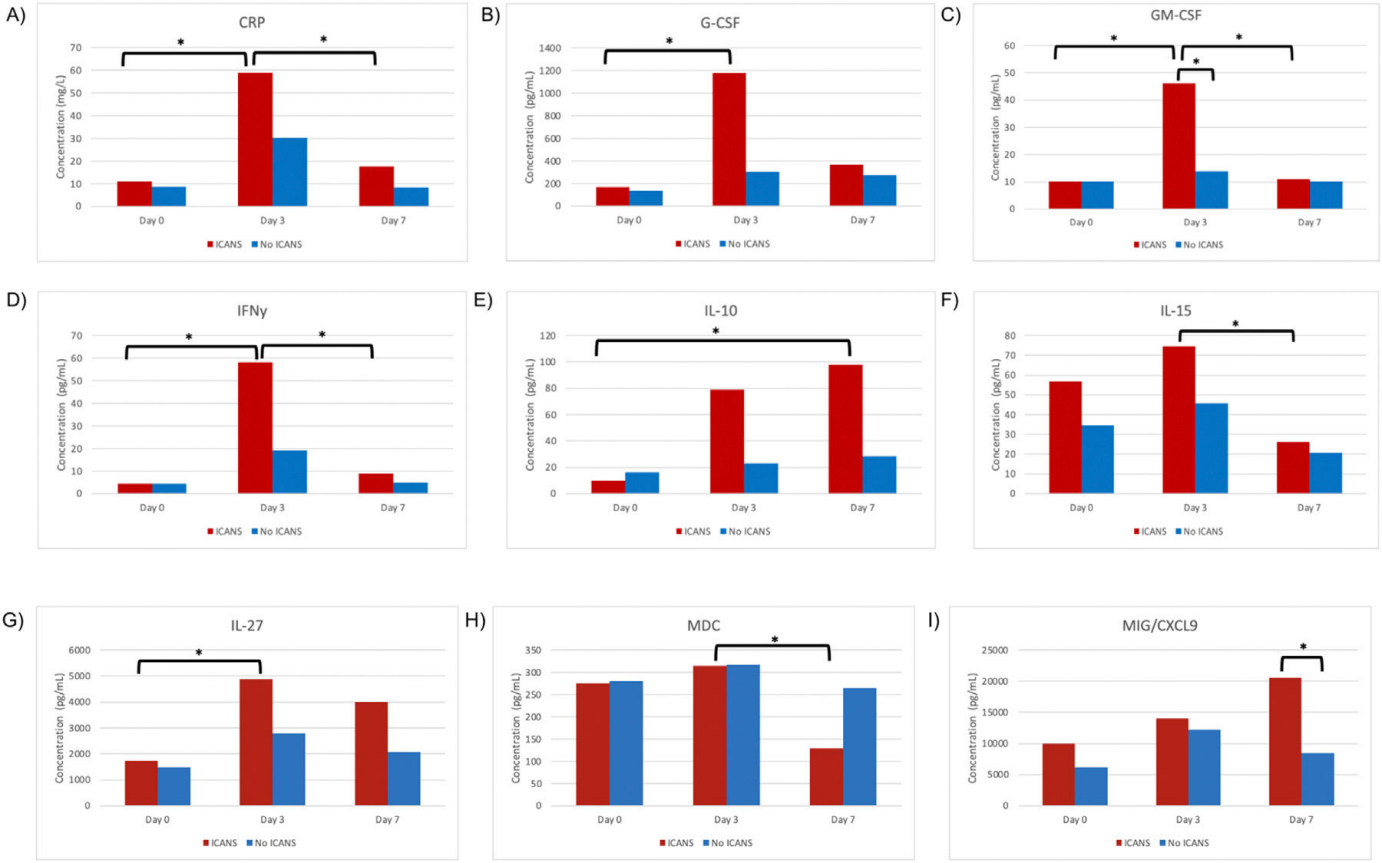
Changes in biomarkers after CAR T-cell therapy in patients with and without ICANS. (A-I) show concentrations of serum cytokines assessed on Day 0 (prior to CAR T-cell infusion), Day +3, and Day +7 following infusion. The red bars correspond to biomarker levels in patients found to have ICANS (*n* = 5) and the blue bars correspond to biomarker levels in patients without ICANS (*n* = 7). Both groups had patients with and without CRS. Asterisks denote significant differences in biomarker levels between 2 groups (*= *P* < .05).

Lastly, serum biomarkers were also measured for all study participants at predefined time points of Day 0 (pre-CAR T-cell infusion), Day +3, and Day +7. Of the 48 human cytokine/growth factors and 6 additional serum markers measured, changes in the levels of 9 biomarkers were associated with the development of ICANS on univariate analysis. Figure 5 and Supplemental Figure S2 display bar graphs demonstrating changes in serum biomarker levels. CRP levels were significantly elevated at Day +3, corresponding to the median onset of ICANS in the study sample, when compared with Day 0 (58.98 vs. 10.96mg/L, *P* = .010) and Day +7 (58.98 vs. 17.5mg/L, *P* = .036). These same significant elevations in CRP on Day +3 were not seen when results were stratified by the presence or absence of CRS. Similarly, GM-CSF levels were significantly elevated at Day +3 when compared with Day 0 (46.18 vs. 10.21 pg/mL, *P* = .010) and Day +7 (46.18 vs. 10.90 pg/mL, *P* = .012), as were IFNγ levels on Day +3 (58.25 vs. 4.54 pg/mL on Day 0, *P* = .010; 58.25 vs. 8.81 pg/mL on Day +7, *P* = .021). Other cytokines with significantly elevated levels on Day +3 in patients with ICANS on univariate analysis include G-CSF, IL-15, IL-27, and MDC. In contrast, IL-10 and MIG/CXCL9 had a delayed increase, with the highest measured mean concentration on Day +7 at 97.94 pg/mL and 20553.36 pg/mL, respectively. For all other measured biomarkers, no significant differences were noted between Days 0, +3, and +7 in patients both with and without ICANS.

## Discussion

This study is the first to our knowledge to utilize abbreviated versions of formal neurocognitive tests to examine early neurologic and cognitive changes in patients during the first 30 days after CAR T-cell therapy. The tests in CART-NS were easily administered by nurses, and their implementation is feasible and reproducible in inpatient and outpatient settings with minimal training. Objective changes in all neurocognitive measures and symptom questionnaires included in CART-NS were seen during Grade 1 and 2 ICANS. When analyzed separately during each day following CAR T-cell infusion, significantly worse performance on the Oral Symbol Digit, Stroop, Trail Making, and Paced Visual Serial Addition Tests was seen in patients who developed ICANS. Changes in these measures were also seen to precede deterioration in the ICE score and physical exam abnormalities associated with ICANS. Among the 3 patients with ICANS who had abnormalities on EEG, a decrease in performance on measures of executive function, attention, and processing speed was observed to correlate with EEG changes consistent with encephalopathy. The tests with the greatest sensitivity in capturing early neurotoxicity assess similar neurocognitive domains, namely executive function, attention, processing speed, and working memory, with performance on some of the measures also being potentially impacted by deficits in speech and language. Deficits in these domains are supported by previous studies identifying cognitive changes after CAR T-cell therapy. ^23^

In our study, brief neurocognitive testing was both sensitive and specific in detecting cognitive changes associated with ICANS, and several tests successfully predicted which patients will develop ICANS after CAR T-cell therapy. When compared to the ICE score, for which changes in most patients were seen between Day +3 and Day +11, a decline in performance for OSD, Stroop, PVSAT, and Connecting Dots was seen in the first 36 hours among patients who subsequently developed ICANS. Of all measures tested, Stroop had the greatest predictive utility for ICANS, while the OSD, Stroop, and PVSAT measures also had a strong predictive utility for ICANS when evaluated together as a combined measure. This finding is comparable to earlier reports in which a multistep command tool administered by physical therapists or nurses was more sensitive than the ICE score in detecting ICANS.^4^ These tests, both alone and when used together early after CAR T-cell infusion, may therefore have future utility in predicting impending ICANS even in the absence of ICE score changes. The earlier detection of impending neurotoxicity has several useful applications in supporting the targeted use of strategies, such as anakinra for ICANS prevention,^24^ as well as minimizing the use of dexamethasone, which can prolong hospitalizations and possibly impair CAR T-cell efficacy when used for extended periods of time.^13,25,26^ Given the ability of ICANS to rapidly progress, earlier detection may also limit deterioration of neurotoxicity to severe Grade 3 or 4 ICANS. Although earlier studies have found the median duration of ICANS to be around 4 days,^27^ differences in performance on OSD, Stroop, and PVSAT persisted beyond clinical resolution of other neurotoxicity signs and symptoms. Notably, patients with ICANS did not have improvements in performance due to practice effects, indicating possible longer-term deficits in memory and learning ability that may be precipitated by acute neurotoxicity or treatment with steroids. These longer-term deficits may not necessarily be captured with existing neurocognitive tools such as the MoCA. For example, patients with ICANS in 1 study had a decline in MoCA performance between 4 and 6 days after CAR T-cell infusion but had recovery to baseline scores by Day +8 after receiving steroids.^14^

On follow-up CART-NS testing at 6 months and 12 months after CAR T-cell therapy, all patients with ICANS in our study had recovery of neurocognitive function to baseline on CART-NS measures. However, 1 patient was noted to have intermittent word-finding difficulty and persistent mood changes that were present at the time of ICANS onset and had delayed recovery. The use of objective neurocognitive measures can thus play a dual role in ensuring that milder cases of neurotoxicity are not missed and in monitoring for the clinical resolution of cognitive symptoms that may not necessitate medical treatment but can impact patient quality of life and indicate a need for follow-up neurocognitive evaluation and intervention.^28^ This is especially important as several studies have revealed an ongoing deterioration in several cognitive domains, including executive function, attention, language, and memory, by patient self-reported measures during the first several years after CAR T-cell therapy.^28–30^ Given that prior reports of cognitive changes following CAR T-cell therapy have relied on physician and nurse observations or self-reported measures,^28–33^ the performance-based tests used in our study eliminate some of the bias and interobserver variability seen with more subjective measures. Future applications of these tests may also facilitate detection of neurotoxicity in CAR T-cell therapy for solid malignancies such as glioblastoma,^34^ where several neurocognitive domains may already be affected at baseline.

The patient and caregiver symptom questionnaires used in this study support previous data on patient-reported common cognitive symptoms after CAR T-cell therapy.^3^ Attentional deficits, difficulty concentrating, new challenges completing activities of daily living, and speech difficulty were the most common symptoms reported by patients and caregivers, all consistent with early symptoms of ICANS. Such mild attentional deficits may be underestimated by the ICE assessment tool due to patient memorization of answers to questions such as counting backwards or naming common objects. Results from the CSQ were more specific than the PSQ, which may reflect patient difficulty in reporting symptoms when encephalopathic or confused and the reporting of symptoms unrelated to ICANS such as anxiety or insomnia. Similar to prior studies, the greatest symptom burden in our cohort was reported during the second week after CAR T-cell therapy and most patients had a gradual return to baseline thereafter.^33^ Future versions of the PSQ and CSQ may therefore be tailored to symptoms that are equally sensitive but more specific for impending progression of ICANS. Simultaneously, self-reported symptom burden may also serve as a useful marker of other neurotoxicity, such as Parkinsonism and motor and neurocognitive toxicity (MNT) seen in patients receiving BCMA CAR T-cell therapy.^35, 36^

Of the serum biomarkers examined in the study, significant elevations in CRP, G-CSF, GM-CSF, IFN γ, IL-10, IL-15, IL-27, and MIG/CXCL-9 were associated with ICANS. While elevated on Day +3, the majority of these cytokines improved to baseline levels by Day +7 with the exception of IL-10 and MIG/CXCL-9. Cytokines previously noted to be sensitive for CRS, such as IL-6, were not significantly associated with ICANS development in contrast to previous reports.^17,18^ However, other factors postulated to be involved in activation (CRP), modulation (IFN γ, IL-10), and proliferation (GM-CSF, IL-15) of the inflammatory response^19^ were all elevated in patients with ICANS. IL-10 is typically thought of as an immunosuppressive modulator, but also has been associated with IFNγ- mediated CD8+ *T* cell cytotoxicity at high levels.^19^ Elevations in LDH and ferritin seen previously in ICANS^37^ were not found in our study population.

There are several important limitations to note in our study. Our study sample was limited to 12 patients and therefore may not be representative of all neurotoxicity and ICANS following CAR T-cell therapy. For instance, practice patterns at our center favored the treatment of Grade 1 ICANS, and there were no reports of Grade 3 or 4 ICANS. All Grade 1–2 ICANS was treated with low-dose, short-term dexamethasone between 3 and 7 days and all Grade 1 CRS was treated with tocilizumab, which may not reflect treatment patterns at other centers. Given the intensity of the testing performed in our study, with 10 measures assessed in each patient every 4 hours to 8 hours, our methodology precluded enrollment of a large sample size. While our sample size limits generalizability, it achieves the intended purpose of establishing the feasibility and validity of various neurocognitive measures for their predictive utility of ICANS development and use in future neurotoxicity screening tools after CAR T-cell therapy. Despite our attempt to minimize practice effects by including 3 alternate testing forms for all measures, most tests still had some degree of practice effects as seen in the positive performance trajectories in patients without ICANS. These practice effects were amplified by the high frequency of testing in our study sample (every 4 hours-8 hours) and can be minimized in the future by limiting use of neurocognitive testing to daily testing during the first several weeks after CAR T-cell infusion followed by weekly or monthly testing thereafter. Notably, the practice effects seen in many of our measures are still significantly lower than the ICE assessment tool and other similar measures, which also have a ceiling effect. Given that all patients with ICANS in the study received treatment with dexamethasone, any effects of steroid treatment on cognition cannot be separated from improvements seen from the resolution of neurotoxicity and neuroinflammation. Lastly, while previously validated versions of each neurocognitive test were used in the selection and design of most of our abbreviated measures, they were not formally validated in a representative age-matched control group. While each patient’s performance was compared to baseline, multicenter testing and further validation of the most promising measures in the future may allow for the use of cutoff scores for varying grades of neurotoxicity.

Future directions should involve adaptations of CART-NS to include the most predictive measures for ICANS and less frequent testing to allow for use within a larger population. For instance, a shorter version of CART-NS with only OSD, Stroop, and PVSAT can be completed in 3 minutes and the remaining measures can be added if patients have an abnormal result. Performance on CART-NS measures did not have significant variability between different times of day, even among patients who developed ICANS, and testing frequency can likely be reduced to once or twice daily in future studies. Furthermore, the neurocognitive tests in CART-NS can also be readily adapted for digital or mobile device testing in the future to facilitate home monitoring of patients enrolled in outpatient CAR T-cell therapy programs and allow standardized collection of data without the need for nurse administration. ^38^

## Conclusion

Brief neurocognitive testing can be feasibly applied for the early detection of ICANS in patients receiving CAR T-cell therapy, predict which patients may go on to develop ICANS in the first 30 days, and overcome some of the limitations of the ICE assessment tool currently in widespread use. Measures evaluating domains of processing speed, attention, executive function and working memory, such as the OSD, Stroop, and PVSAT were both sensitive and specific in predicting ICANS development and can be combined with symptom questionnaires to capture the full spectrum of neurotoxicity symptoms. Serum biomarkers associated with neuroinflammation, including CRP, G-CSF, GM-CSF, IFNγ, IL-10, and IL-15, were also associated with the development of ICANS. Ultimately, systematic use of these measures may allow earlier intervention to minimize or prevent toxicity, mitigate the need for prolonged steroids, and optimize overall outcomes following CAR T-cell therapy.

## Supplementary Material

Suppl Material

Supplementary material associated with this article can be found, in the online version, at doi:10.1016/j.clml.2024.12.011.

## Figures and Tables

**Table 1:** Mapping of Neurotoxicity Signs and Symptoms to Neurocognitive Domains and Measures

Neurotoxicity Signs or Symptom^Refs^	Neurocognitive Domain	Neurocognitive Measure

Dysarthria, ^3,5,6^	Speech and Language	Oral Symbol Digit, Verbal Fluency
Aphasia^2–6,8–11^	Speech and Language	Oral Symbol Digit, Verbal Fluency
Bradyphrenia,^2,3,7,8^	Processing Speed	Oral Symbol Digit, Trail Making, Stroop, PVSAT
Tremor ^2,5–11^	Fine Motor	Finger Tapping, Connecting Dots
Dyskinesia^3,9^	Fine Motor	Finger Tapping, Connecting Dots
Ideomotor apraxia,^2,3,5,6^	Fine Motor	Finger Tapping, Connecting Dots
Confusion and disorientation (encephalopathy) ^2–11^	Executive Function, Attention	Trail Making, Stroop, PVSAT, Patient and Caregiver Symptom Questionnaire
Memory disorders^2–5,8^	Working Memory	PVSAT
Dyscalculia,^2,3^	Processing Speed, Working Memory	PVSAT
Distractibility ^2–5,7–9^	Executive Function, Attention	Trail Making, Stroop, PVSAT
Visual agnosia	Visual-spatial Integration	Intersecting Figures
Headache,^2,3,5–9,11^ and neck stiffness^3,5^	Meningismus	Patient Symptom Questionnaire
Impulsivity, hallucinations, agitation,^2,4,6,10^ restlessness,^2,3^ apathy, ^6^ depression,^8,9^ anxiety^2,9,11^	Personality, Mood	Patient and Caregiver Symptom Questionnaire

**Table 2:** Participant Characteristics and Demographics

Demographic Characteristics	Number of Study Participants (%) *n* = 12

Age	
*<* 50	2 (16.7%)
50–59	0 (0%)
60–69	6 (50%)
≥ 70	4 (33.3%)
Gender	
Male	8 (75%)
Female	4 (25%)
Handedness	
Left	2 (16.7%)
Right	10 (83%)
Baseline eGFR	
> 90	8 (75%)
60 to 89	2 (16.7%)
45 to 59	2 (16.7%)
CAR T-cell Agent Received	
Axicabtagene ciloluecel (CD19-directed)	4 (33.3%)
Tisagenlecleucel (CD19-directed)	4 (33.3%)
Idecabtagene vicleucel (BCMA-directed)	4 (33.3%)
Lymphodepletion Regimen	
Fludarabine/Cyclophosphamide	12 (100%)
None	0 (0%)
Primary Malignancy at Time of Infusion	
Diffuse Large B-cell Lymphoma	5 (41.7%)
Mantle Cell Lymphoma	0 (0%)
Follicular Lymphoma	3 (25%)
Multiple Myeloma	4 (33.3%)
Disease Stage	
Stage I	0 (0%)
Stage II	4 (33.3%)
Stage III	3 (25%)
Stage IV	5 (41.7%)
Prior Lines of Therapy	
1	0 (0%)
2	2 (16.7%)
3	4 (33.3%)
4 or More	6 (50%)
Bridging Therapy	
Yes	10 (83.3%)
No	2 (16.7%)
Disease Status Before CAR T-cell Therapy	
Progressive Disease	4 (33.3%)
Stable Disease	1 (8.3%)
Partial Remission	6 (50%)
Complete Remission	1 (8.3%)
Peak CRP After CAR T-cell Therapy*	
≥ 100	3 (25%)
50–99	4 (33.3%)
5–49	5 (41.7%)
*<* 5 (Normal)	0 (0%)
Maximum Grade of CRS*	
None	5 (41.7%)
Grade 1	3 (25%)
Grade 2	4 (33.3%)
Grade 3	0 (0%)
Grade 4	0 (0%)
Lowest ICE Score*	
10	8 (66.7%)
9	2 (16.7%)
8	2 (16.7%)
Less Than 8	0 (0%)
Maximum Grade of ICANS*	
None	7 (58.3%)
Grade 1	2 (16.7%)
Grade 2	3 (25%)
Grade 3	0 (0%)
Grade 4	0 (0%)

* As measured between Day 0 through +30 after CAR T-cell product infusion.

**Table 3:** Overall Performance on CART-NS Neurocognitive Measures

Test Characteristics		Mean Score (% Change in Performance From Baseline)							

Test Name	Scoring Measure	ICANS Present	ICANS Absent	*P* Value	Cohen’s d	ICE Change	ICE Change	*P* Value	Cohen’s d
		(*n* = 107)	(*n* = 718)			Present (*n* = 28)	Absent (*n* = 797)		
OSD	# correct in 20 s	10.8 (−12%)	12.8 (+13%)	< .001	0.76	7.9 (−35%)	12.7 (+12%)	< .001	1.84
Trails*	Seconds to completion	55.0 (−29%)	29.4 (+17%)	< .001	0.92	109.1 (−117%)	30.0 (+15%)	< .001	3.11
Stroop	# correct in 20 s	13.5 (−21%)	18.3 (+15%)	< .001	0.80	9.6 (−36%)	18.0 (+12%)	< .001	1.39
PVSAT	# correct in 20 s	13.4 (+6%)	14.5 (+24%)	.014	0.24	10.5 (−5%)	14.5 (+23%)	< .001	0.85
Finger Tapping*	Seconds to completion								
R		14.5 (+3%)	14.4 (+8%)	.827	0.02	16.2 (+8%)	14.3 (+7%)	.009	0.36
L		15.3 (−4%)	14.0 (+2%)	.004	0.29	17.5 (−4%)	14.0 (+2%)	< .001	0.72
Connecting Dots*	Seconds to completion								
R		17.9 (−25%)	16.4 (−12%)	.002	0.34	16.3 (−21%)	16.6 (−14%)	.658	0.07
L		22.2 (−35%)	20.3 (−18%)	.005	0.31	21.1 (−34%)	20.6 (−20%)	.707	0.09
Intersecting Figures	Accuracy out of 6 points	5.9 (−1%)	6.0 (+1%)	.014	0.38	5.9 (−2%)	6.0 (+1%)	.096	0.61
Verbal Fluency	# words in 1 min	10.1 (+0%)	14.6 (+17%)	< .001	0.74	8.1 (+0%)	14.3 (+16%)	< .001	1.00
PSQ	Score out of 18	1.4	0.5	< .001	0.79	1.5	0.6	.111	0.75
CSQ	Score out of 12	0.6	0	< .001	1.37	1.7	0.1	< .001	4.43

* A decrease in performance on these measures indicates a longer time to task completion.

## Clinical Practice Points

- Immune effector cell-associated neurotoxicity syndrome (ICANS) can be a severe, life-threatening toxicity following CAR T-cell therapy and is characterized by a wide range of clinical and neuropsychiatric signs/symptoms, not all of which are fully captured using existing measures such as the immune effector cell-associated encephalopathy (ICE) score.
- Predictive markers of neurotoxicity are necessary to determine those at greatest risk for severe ICANS and the patients likely to experience long-lasting neurocognitive effects.
- We report in this study a novel application of formal neurocognitive testing that can be readily adapted for use in patients immediately following CAR T-cell therapy.
- Neurocognitive tests in our tool are feasibly administered by nurses in the inpatient setting and can detect early and delayed neurotoxicity that is not always captured by the ICE score or reflected in consensus ICANS grading criteria.
- Several measures showed predictive utility in identifying patients who will later go on to develop ICANS, which could allow for prophylaxis and early intervention to improve overall outcomes after CAR T-cell therapy.
