# Supplementary material for: Novel Neurocognitive Testing Tool for Early Neurotoxicity Detection Following Anti-CD19 and Anti-BCMA Chimeric Antigen Receptor (CAR) T-cell Therapy: A Pilot Study
Source: Clin Lymphoma Myeloma Leuk. Author manuscript; Available in PMC 2026 Apr 16. (PMC13086173; doi:10.1016/j.clml.2024.12.011)
Supplement: Suppl Material [file NIHMS2142620-supplement-Suppl_Material.pdf]

SUPPLEMENTARY MATERIAL

Novel Neurocognitive Testing Tool for Early Neurotoxicity Detection Following Anti-CD19 and Anti-BCMA Chimeric Antigen Receptor (CAR) T-cell Therapy: A Pilot Study

Arvind Suresh, Heather A. Wishart, Maeen N. Arslan, Raphael A. Lizcano, Parth S. Shah, Swaroopa PonnamReddy, Christi Ann Hayes, Bryce Jacobson, Grant Moncrief, Pablo Martinez-Camblor, Amy M. Chan, Kenneth R. Meehan, John M. Hill Jr.

To re-use, reproduce, or distribute any supplementary material, please contact the corresponding author:

Arvind Suresh

Department of Medicine, University of California, San Francisco

505 Parnassus Avenue, M-1480, San Francisco, CA 94143

Email: arvind.suresh@ucsf.edu

Supplementary Figures

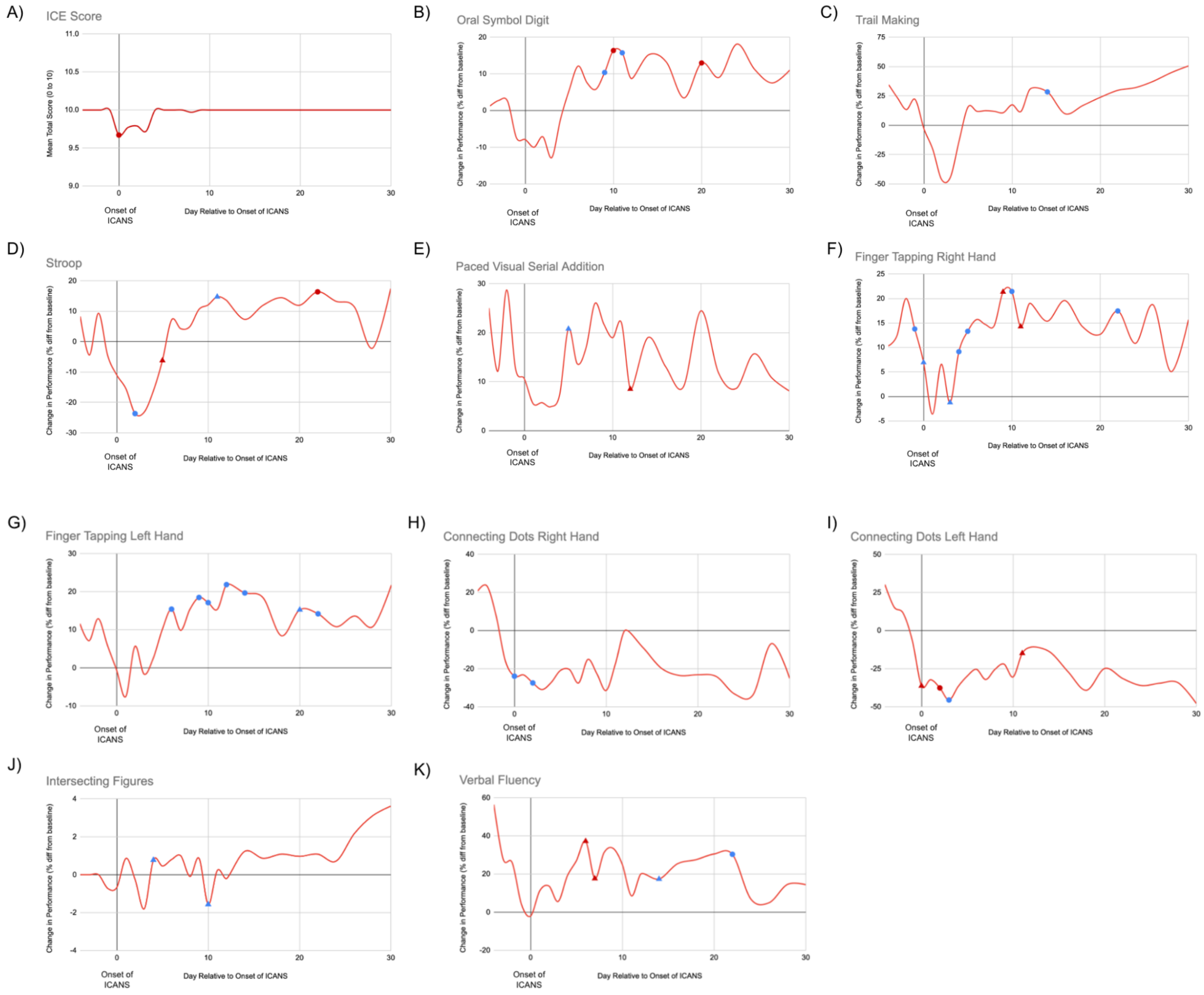

Figure S1. Changes in Neurocognitive Measures Relative to Onset of Neurotoxicity in Patients with ICANS.

Figure S1A demonstrates changes in the ICE score on a scale of 0-10 relative to the onset of ICANS in the study sample.

For **Figures S1B-K,** scores for each subsequent measure are reported as mean percent change from baseline scores for each day relative to the onset of neurotoxicity. Days to the left of 0 are prior to the onset of ICANS and days to the right of 0 are after the onset of ICANS. Scores above 0 indicate better performance and scores below 0 indicate worse performance compared with baseline. Days with a significant change in performance compared to baseline are indicated with red circles ($p < 0.05$) or blue circles ($p < 0.10$). Days with a significant change in performance compared to the previous day are indicated with red triangles ($p < 0.05$) or blue triangles ($p < 0.10$).

**All patients except one in each sub-group (ICANS and non-ICANS) were right-hand dominant.*

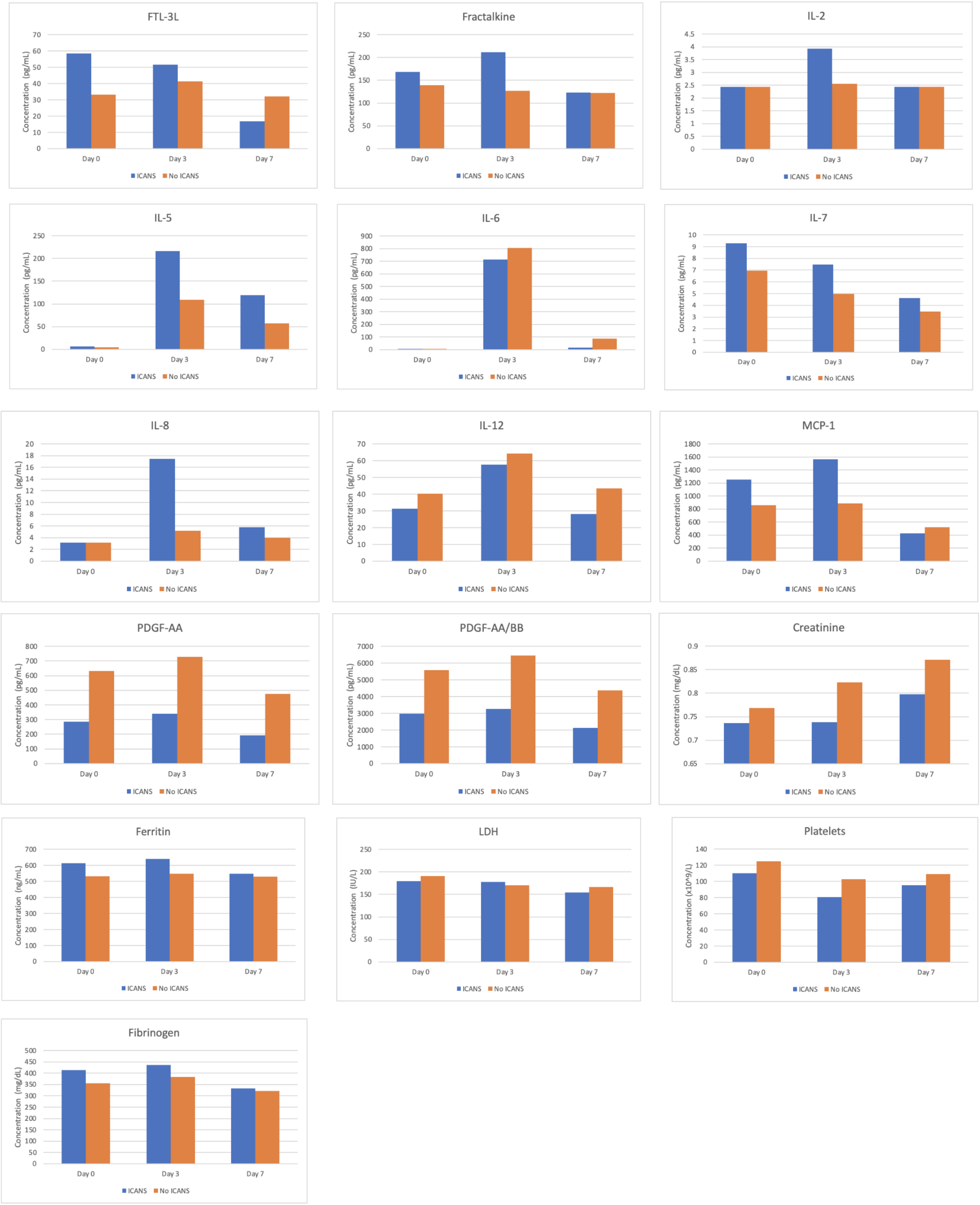

Figure S2. Additional Biomarkers Evaluated After CAR T-cell Therapy in Patients with and without ICANS. Bar graphs show concentrations of serum cytokines assessed on Day 0 (prior to CAR T-cell infusion), Day +3, and Day +7 following infusion. The blue bars correspond to biomarker levels in patients found to have ICANS (n=5) and the orange bars correspond to biomarker levels in patients without ICANS (n=7). Both groups had patients with and without CRS.

Supplementary Appendices

All appendices start on the following page

Appendix S3. Descriptions of Neurocognitive Tests in CART-NS

Appendix S4. CART-NS Patient and Caregiver Symptom Questionnaire

APPENDIX S3:

CAR-T Neurotoxicity Screener (CART-NS)

Descriptions of Neurocognitive Tests

Oral Symbol Digit: The patient is shown a key containing a series of symbols, and each symbol is paired with a single digit number. Below the key is a series of symbols and the participant is instructed to say aloud the number that corresponds with that symbol. The patient has 20 seconds to say as many as they can. The score is total number of correct associations in 20 seconds.

Trails Test: The patient is shown an array of numbers and letters randomly distributed on a page. The patient is asked to draw a line from 1 to A, 2 to B, and so on until G, which is the end point. If the patient makes a mistake, the examiner stops them and asks them to correct it before going on. The score is time in seconds to complete the entire test accurately.

Stroop Test: The patient is shown a page with names of colors printed in a non-corresponding color of ink, e.g., the word green printed in blue ink. They are instructed to say aloud the color of the ink and not read the word. The score is total number correct colors stated in 20 seconds.

Paced Visual Serial Addition Test: The patient is shown multiple rows of numbers of page. For each row, the patient is instructed to add each number on the page to the prior one. The score is total number of correct additions in 20 seconds.

Finger Tapping: The patient is shown a page that has four dots, and they are asked to position the fingers of their right hand comfortably over the dots and place the heel of their hand on the page. Then they are instructed to tap the dots one at a time in order, moving from the 2nd to 5th finger . The test always starts with the right hand regardless of whether the person is right-hand dominant. The procedure is then repeated with the left hand. The score is time in seconds to complete ten full sets of tapping with each hand.

Connecting Dots: The patient is shown a page with a column of X's on the left side and another column of X's on the right side. They are instructed to draw a straight line from the middle of each X on the left to the middle of the corresponding X on the right, and to avoid having the lines touch each other. This procedure is completed first with the right hand and then with the left, regardless of whether the person I right-hand dominant. The score is the total time in seconds to connect all the X's with each hand.

Intersecting Figures: The patient is shown two intersecting figures (either pentagons or hexagons) and asked to copy them as accurately as possible. Their drawing is scored on a 0 to 6 scale based on specific written criteria. A score of 6 indicates perfectly copied drawing, while points are deducted for errors that are made.

Verbal Fluency: The examiner says a letter of the alphabet and the patient has 60 seconds to say aloud as many different words as they can that start with that letter. The score is total number of named words starting with the correct letter. Repetitions are not counted and Proper nouns are allowed.

APPENDIX S4:

CAR-T Neurotoxicity Screener (CART-NS)

SYMPTOM QUESTIONNAIRES

Patient Symptom Questionnaire

1. Do you have a new headache? (Check One)

☐ 3 – that is worse when you bend your neck
☐ 2 – that is worse when you lay down such that you have to sit up
☐ 1 – that is constant and not dependent on position
☐ 0 – not at all

2. Do you have new dizziness or unsteadiness? (Check One)

☐ 3 – Very much indeed. I can feel suddenly pulled to one side or another, and fall.
☐ 2 – Quite a lot. I am unsteady. I feel like I am on a moving boat.
☐ 1 – Not very much. I feel a little lightheaded but I can walk without falling
☐ 0 – not at all

3. Do you have new trouble with talking? (Check One)

☐ 3 – Very much indeed. I can barely talk. The words just will not come out.
☐ 2 – Quite a lot. My speech is like 'stop and go'. I keep losing words.
☐ 1 – Not very much. Sometimes it is harder to come up with names of things.
☐ 0 – Not at all

4. Think about doing your usual daily activities. Do you have new difficulty concentrating? (Check One)

☐ 3 – Very much indeed. I stopped the activity. I just cannot do it right.
☐ 2 – Quite a lot. I keep forgetting what I was just doing and have to start over again.
☐ 1 – Not very much. It is hard to focus sometimes.
☐ 0 – Not at all

If you checked > 0 on the previous question, please elaborate on what activity and what's different:

____
____
____
____
____

5. Do you have new anxiety? (Check One)

☐ 3 – Very much indeed. I feel tense or 'wound up' most of the time. My mind is constantly switching from one thing to another.
☐ 2 – Quite a lot. I feel tense most of the time. I cannot sit at ease and feel relaxed.
☐ 1 -- Not very much. I have occasional worries, but not very often.
☐ 0 – Not at all

6. Do you have new depression or loss of interest in activities? (Check One)

☐ 3 – Very much indeed. I feel "down" all the time and have no interest in any activities.
☐ 2 – Quite a lot. I have difficulty feeling hopeful and my energy is low for most of the day.
☐ 1 -- Not very much. I occasionally feel "down" and/or my enjoyment of usual daily activities has decreased.
☐ 0 – Not at all

7. Do you have new hallucinations, such as: (Check box if Yes)

☐ Smells that no one else can smell
☐ Flashing lights, or seeing shapes or things that are not there
☐ Voices that no one else can hear, or sounds that fade away
☐ Strange taste in your mouth that is not there
☐ Numbness, vibration or crawling sensation that move from one part of your body to another

8. Thank you for completing these tests. Please make any notes here that you think are important for the study team to know about this testing session, including your observations and any difficulties you encountered.

____
____
____
____
____
____
____
____
____
____

CAREGIVER SYMPTOM QUESTIONNAIRE

1. Do you notice new restlessness or irritability in the patient? (Check One)

☐ 3 – Very much indeed. S/he is restless. Constantly switching from doing one thing to another. Easily irritated when addressed.
☐ 2 – Quite a lot. S/he looks tense most of the time. S/he is frequently fidgeting. There can be some uncharacteristic speech or outbursts.
☐ 1 -- Not very much. S/he intermittently looks impatient or irritated. However, s/he can sit at ease for most of the time.
☐ 0 – Not at all

2. Do you notice changes in mood or interest in activities? (Check One)

☐ 3 – Very much indeed. Can have frequent crying or disruptive vocalizations, shows no interest in any activities.
☐ 2 – Quite a lot. Withdrawn with occasional emotional outbursts, still shows some interest in activities.
☐ 1 -- Not very much. Less animated than usual; initiates fewer conversations.
☐ 0 – Not at all

3. Do you notice the patient having any new trouble talking? (Check One)

☐ 3 – Very much indeed. The patient barely speaks. The words just will not come out *OR* s/he seems to stare in space most of the time.
☐ 2 – Quite a lot. The patient's speech is like 'stop and go'. S/he keeps losing words. It takes a long time for him/her to answer.
☐ 1 – Not very much. It is harder for him/her to come up with names of things. S/he seems more distractible, and occasionally loses train of thought.
☐ 0 – Not at all

4. Do you notice the patient having new confusion in usual activities of daily living, for which their ability or performance has changed? (Check One)

☐ 3 – Very much indeed. S/he has stopped doing usual activities (please list below). S/he just cannot do it right. May stare at task with blank look.
☐ 2 – Quite a lot. S/he seems to keep forgetting what to do. Keeps restarting the task and makes mistakes along the way.
☐ 1 – Not very much. There is some fumbling with tasks. S/he looks unfocused and performs tasks slower or more messy than usual.
☐ 0 – Not at all

If you checked > 0 on the previous question, please elaborate on what activity and what's different: (Write Below)

____
____
____

6. Are there any other observations that are concerning, including change in cognitive ability, physical agility, mood, personality or just uncharacteristic of him / her? (Write Below)

____
____
____
____

7. Please use the space below to write anything about this testing session that the study team should know (i.e. observations, any difficulties or interruptions, inability to perform certain tests, etc.).

____
____
____
____
____
____
